# Polyphenols in Farm Animals: Source of Reproductive Gain or Waste?

**DOI:** 10.3390/antiox9101023

**Published:** 2020-10-21

**Authors:** Nesrein M. Hashem, Antonio Gonzalez-Bulnes, Jesus Simal-Gandara

**Affiliations:** 1 Department of Animal and Fish Production, Faculty of Agriculture (El-Shatby), Alexandria University, Alexandria 21545, Egypt; 2 Departamento de Reproducción Animal, INIA, Avda, Puerta de Hierro s/n., 28040 Madrid, Spain; bulnes@inia.es; 3 Departamento de Produccion y Sanidad Animal, Facultad de Veterinaria, Universidad Cardenal Herrera-CEU, CEU Universities, C/ Tirant lo Blanc, 7, Alfara del Patriarca, 46115 Valencia, Spain; 4 Nutrition and Bromatology Group, Department of Analytical Chemistry and Food Science, Faculty of Food Science and Technology, University of Vigo-Ourense Campus, E-32004 Ourense, Spain; jsimal@uvigo.es

**Keywords:** plant polyphenols, antioxidant, reproduction, farm animals, hormones

## Abstract

Reproduction is a complex process that is substantially affected by environmental cues, specifically feed/diet and its components. Farm animals as herbivorous animals are exposed to a large amount of polyphenols present in their natural feeding system, in alternative feed resources (shrubs, trees, and agro-industrial byproducts), and in polyphenol-enriched additives. Such exposure has increased because of the well-known antioxidant properties of polyphenols. However, to date, the argumentation around the impacts of polyphenols on reproductive events is debatable. Accordingly, the intensive inclusion of polyphenols in the diets of breeding animals and in media for assisted reproductive techniques needs further investigation, avoiding any source of reproductive waste and achieving maximum benefits. This review illustrates recent findings connecting dietary polyphenols consumption from different sources (conventional and unconventional feeds) with the reproductive performance of farm animals, underpinned by the findings of in vitro studies in this field. This update will help in formulating proper diets, optimizing the introduction of new plant species, and feed additives for improving reproductive function, avoiding possible reproductive wastes and maximizing possible benefits.

## 1. Introduction

The livestock production is currently challenged by environmental issues and consumers’ attitude and lifestyle. The most important among these considerations are the limitation of natural resources (land and water), climate change (global warming, desertification and greenhouse gases), and consumers’ demand for organic and functional products fulfilling the principles of animal welfare. Hence, foodstuffs for livestock are more and more focused to the use of natural autochthonous resources rather than the traditional cultivation of foreign plants which are highly demanding of water and soil quality. Moreover, there is increasing knowledge that plants and plant products are not only food but can also be considered nutraceuticals due to their unique chemical composition, specifically secondary metabolites with potential biological benefits on animal performance and quality of animal products. Among the secondary metabolites of plants, polyphenols have gained considerable attention in the last few years due to their abundance in different plants and desirable biological activities. Many studies have confirmed antioxidant, anti-inflammatory, metabolism- and immunity-modulatory activities of polyphenols, but also antihelminthic, antimethanogenic and antimicrobial effects [1], which are of particular importance in livestock production [2,3]. These properties encourage research and awareness for the use of these secondary metabolites as natural tools to improve animal performance and animal product quality.

In extensive livestock production, characterized by low incomes and the limitation of feed resources, it is necessary to look for alternative and available foodstuffs such as shrubs, trees, and agro-industrial byproducts. These products have been found to naturally contain substantial amounts of polyphenols and are more and more used for animal nutrition, having in mind the positive effects of polyphenols on animal growth, performance and health, on adequacy of nutrients utilization, on mitigation of methane emission, and improvement of the quality of animal products [2,4,5,6,7,8,9].

However, there is also evidence about the negative effects for animal homeostasis, especially affecting reproductive events. Early evidence, in 1940, showed that feeding sheep with *Trifolium (T.) pratense* L. (red clover; which is rich in isoflavones, a subclass of polyphenols) is related to high infertility rates by disrupting embryo survival and fetal development; the same was confirmed in cows about 40 years later [10]. Afterwards, the studies on the benefits/risks of polyphenols supplementation are scarce and controversial [11,12,13] and most of the studies on the utilization of polyphenols in the diets of farm animals have ignored the effects of polyphenols on reproductive performance.

Actually, the balance between the risks and benefits from the use of polyphenols may be in favor of its use in growing animals bred for meat production, as the negative effects, if present, will be ended by the end of the productive cycle of the animal. However, the benefits may be more questionable in breeding animals; mainly because improper polyphenols intake may not only affect the reproductive performance of the parents but may also induce effects on the progeny, due to epigenetic changes affecting gene expression/programming and thus the future performance and health/disease status of the offspring [14]. These epigenetic effects may occur not only by parenteral exposure, but also through application of assisted reproductive techniques (ARTs) in which polyphenols, aiming its antioxidant or antibacterial effects, are included in the media of cryopreserved or cultured gametes/embryos [3,15,16].

In view of these considerations, the use of polyphenols, both in vivo and in vitro, needs further investigation and caution prior to its systematic application in practice. The present review aims to illustrate recent findings connecting both dietary polyphenols consumption from different sources (conventional feed stuffs, alternative feed resources, and feed additives) and reproductive performance of farm animals, but also the available information on the use of the compounds for ARTs. This update aims to report the current knowledge on the field and set the scenario for subsequent studies focused on formulating proper diets and introducing new plant species/materials and feed additives in an adequate approach to the reproductive function/stage of the animals, avoiding possible reproductive risks and maximizing possible benefits.

## 2. Polyphenols Sources in Animal Diets

Most farm animals, as herbivorous animals, are naturally exposed to substantial amounts of polyphenols as a part of their usual feed system. Many seeds and roughage used in farm animals’ feeding contain polyphenols with different concentrations and forms, conferring each plant its unique polyphenol profile (Table 1). For example, formononetin, daidzein, genistein, and biochanin A are the major polyphenols in *Trifolium* (*T*.) *subterraneum* [17] and *T. pratense* [11,18] containing isoflavones, which account for up to 5% of dry matter [19], while coumestans are the major polyphenols in *Medicago sativa* [18] and *Melilotus albus* [20].

In traditional farming systems, *Glycine max* (soybean: [44,45]), *Linum usitatissimum* (linseed [44]), and clover species such as *T. subterraneum* [14], *T. pratense* L. (red clover [11,18]), and *Trifolium alexandrinum* (Berseem clover: [13]) are the most common sources of polyphenols in animal diets. Due to the limitation of traditional animal feed resources, many feed alternatives such as shrubs and trees are being introduced as new feedstuff. Interestingly, these feed alternatives have been found to contain substantial amounts of polyphenols. In these plants, tannins can be found in almost 80% of woody perennial dicotyledonous species and 15% of annual and herbaceous perennial dicotyledons such as trees, shrubs, legumes, herbs, and cereal grains [6,7]. Many of them are used as animal feeds such as *Acacia* [31], *Dichrostachys, Dorycnium, Hedysarum, Leucaena, Lotus* [28], *Onobrychis, Populus, Rumex* and *Salix* [30], and *Quercus robur* with prevalence condensed tannins accounting for up to 20% of the dry matter [46]. 

Inclusion of agro-industrial byproducts in farm animal diets is parallel to the calls to use polyphenol-based feed additives due to their claimed antioxidant activity and beneficial effects on farm animal performance and animal product quality (meat and milk). Agro-industrial byproducts, such as fruit and vegetable industrial byproducts, are being introduced as unconventional feed stuff (Table 1); among them, the most used are *Vitis vinifera* (grape), *Punica granatum* (pomegranate), *Olea europaea* L. (olive), *Camellia sinensis* (green tea), *Solanum lycopersicum* (tomato) and *Citrus aurantifolia* (citrus) residues [7]. Each one of these byproducts has a unique polyphenol profile (Table 1). In this regard, *Vitis vinifera* residues contain flavonoids (anthocyanins or quercetin), stilbenes (resveratrol), and tannins [47], while *Camellia sinensis* residues are rich in flavonoids (catechins; [48]), and *Olea europaea* L. residues are abundant in phenolic acids (Hydroxytyrosol and tyrosol; [35]).

These facts lead to the conclusion that farm animals are expected to massively consume dietary polyphenols in their daily ration intake during their lifecycle. Accordingly, assurance of the safety of these plant materials on the performance of farm animals, specifically, reproductive performance is a crucial aim to avoid any possible reproductive waste and consequence loss in economic efficiency of the farm. 

## 3. Intake, Absorption, Bioavailability and Bioactivity of Polyphenols

**Figure 1:**
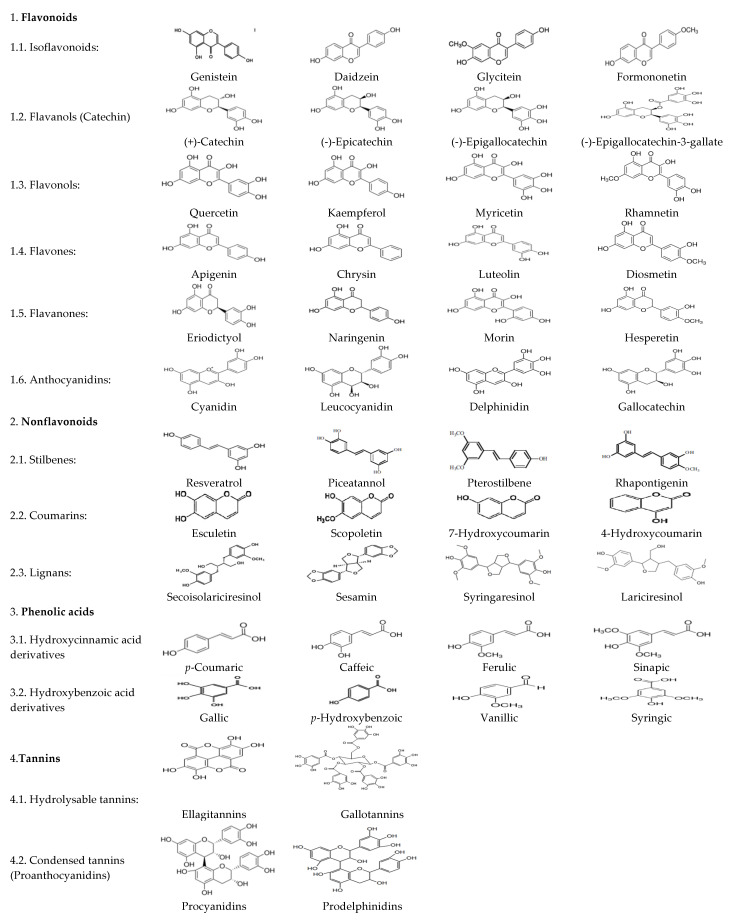
Classes and examples of chemical structures of different plant polyphenols.

Plant polyphenols are secondary metabolites, crucially contributing to plant defense mechanisms against pathogen and insect attacks, herbivorous, wounding, solar radiation, and other stressful conditions [49]. To date, about 8000 molecules have been identified as polyphenols and classified according to their chemical structures in flavonoids, nonflavonoids, phenolic acids, and tannins (Figure 1) [49,50,51,52]. Flavonoids and phenolic acids are the most abundant and account for around one- and two-thirds of total polyphenols, respectively. Plants polyphenols are conjugated with sugar residues and/or other phenols, organic acids, amines, and lipids [49]. After intake, less than 5–10% of plant polyphenols are absorbed through the enterocytes, either by passive diffusion or by selective transportations, but only if they are in the form of aglycones or simple glycosides. These facts compromise its kinetics through the digestive tract and its availability for later metabolization through normal biological pathways, so bioavailability of polyphenols may be insufficient to induce significant biological effects in most of the cases [53]. Different studies have confirmed polyphenol bioavailability and bioactivity, either directly by measuring their concentrations or their metabolites in plasma and urine, or indirectly by evidencing increased antioxidant capacity of plasma or tissues [1]. 

The bioavailability of polyphenols has been confirmed in different reproductive organs including reproductive centers in the brain (hypothalamus and hypophysis), testis, ovary, uterus placenta, and fetus [51,54,55], which confirms the ability of polyphenols to pass different blood barriers of the reproductive organs and therefore to presumptively influence their physiological functions. The bioavailability of polyphenols through the reproductive organs, however, depends on several factors such as the type of polyphenol, the selective transport of the tissue, and the physiological status of the animal. The tissue selectivity to different polyphenols was evidenced by comparing catechins concentrations at maternal blood, placenta, and fetus in rats after receiving epicatechin and epigallocatechin-3-gallate (EGCG) in the form of *Camellia sinensis* (green tea) extract [56]. Maternal plasma showed 10-fold higher levels of catechins than placenta and 50–100-fold higher than the fetus. However, it is also important to highlight that placenta and fetus showed low epicatechin and high EGCG levels, which suggest that epicatechin, opposite to EGCG, is well absorbed and distributed in the maternal circulation but not in the conceptus. The physiological status of the animal is also critical for polyphenols bioavailability, as confirmed by studies comparing concentrations of isoflavones and their metabolites in the blood plasma of late- or early-pregnant heifers in which significantly higher concentrations were found in early pregnant-heifers [54]. Health status and changes in the immune and pro/anti-inflammatory status are also crucial factors affecting polyphenol bioavailability. In this sense, a proinflammatory status, such as occurring during mastitis and metritis, has been found to increase the bioavailability of isoflavones and their metabolites [54]. 

The bioactivity of polyphenols depends moreover on several factors including the polyphenol molecular weight, its conjugation with other derivatives, its hydrolysation by digestive tract enzymes (stomach and intestine), the action of rumen or cecal microflora, and the binding affinity for blood plasma proteins [57].

The action of enzymes from the digestive tract and microflora led to form new polyphenol metabolites with different biological activity than original compounds. For instance, lignans (e.g.,: matairesinol, lariciresinol and secoisolariciresinol) can be metabolized by gastrointestinal bacteria to more potent estrogenic “mammalian lignans” (enterodiol and enterolactone [20]). For example, isoflavones such as genistein and biochanin A are converted to a nonestrogenic metabolite (p-ethyl phenol) whilst formononetin and daidzein are converted to a potent estrogenic metabolite (equol). The effects caused by equol, the formononetin and daidzein metabolite, are the most known example that highlights the importance of weighing the use of polyphenols in animal production; the reproductive disorders observed in sheep herds fed with *T. pratense* L. (named as clover disease [20]).

## 4. Polyphenols and In Vivo Reproductive Events

Polyphenols, as previously mentioned, have confirmed a huge amount of beneficial actions (antioxidant, anti-inflammatory, antimicrobial, antihelminthic, antimethanogenic and metabolism- and immunity-modulatory activities [1]), but also other nondesirable effects. Polyphenols have been recognized as the main environmental-disrupting chemicals that might alter mammalian hormonal balance and reproductive functions in both males [44] and females [58,59].

The unique chemical structure of polyphenols and its similarity to the chemical structure of mammalian estrogens enables them to possess hormone-like effects through binding and activating estrogen receptors (ERs: ERα and ERβ), which result in estrogen-agonistic or antagonistic effects. The binding affinity of polyphenols for ERs is determined by their chemical structure, with the presence of a phenolic ring being responsible for binding to ERs, molecular weight, and optimal hydroxylation pattern [60,61]. Overall, binding affinity of polyphenols is always lower than the natural ligand estradiol (E_2_) [20] and the estrogen-agonistic or antagonistic effects are modulated by the fact that, unlike E_2_, which binds with a similar affinity to both subtypes of ERs, polyphenols have different binding affinity for the two subtypes of ERs (excepting resveratrol, with comparable affinity to both subtypes of ERs). In this regard, genistein, daidzein, equol, and coumestrol show a higher affinity for ERβ than for ERα, with coumestrol estrogenic activity being around 15-fold higher than isoflavones, genistein, biochanin A, and formononetin [20]. Contrarily, 8-prenylnaringenin has around 100-times higher affinity to ERα, but weaker for ERβ than genistein [62]. Thus, different affinities of polyphenols toward both subtypes of ERs and different distribution of ERs among reproductive tissues greatly affect the final result of exposure to polyphenols [63]. Through this mode of action, polyphenols may intervene in the regulation of all reproductive events through hormone modulation of neurohormones (gonadotropin-releasing hormone, GnRH, and oxytocin), gonadotropins (luteinizing hormone, LH, and follicle-stimulating hormone FSH), steroids (E_2_, progesterone, P_4_, and testosterone, T), and prostaglandins. 

Polyphenols may also control steroid function by binding or inactivating sex production enzymes, such as P450 aromatase, 5α-reductase, 17β-hydroxysteroid dehydrogenase (17β-OHDH), topoisomerases, and tyrosine kinases. In addition, polyphones can alter sex hormone binding globulin (SHBG) affinity, and thus levels of active steroids in blood circulation [57]. Finally, polyphenols can also affect the reproductive functions through controlling the expression of genes or the activity of enzymes that contribute to the regulation of reproductive events: (1) activity of enzymes controlling DNA replication (topoisomerases I and II) and extracellular signal regulated kinases; (2) antioxidant and inflammatory molecular pathways; (3) cellular apoptotic and proliferation pathways; (4) modification of the expression of genes related to synthesis of angiogenesis factors in different reproductive tissue; (5) epigenetic mechanisms involving both hypermethylation and hypomethylation; (6) regulation of metabolic hormone signals such as growth hormones, insulin-like growth factors, and triiodothyronine; and (7) modification of the expression of genes associated with fatty acids metabolism [13,55,57,64,65].

**Figure 2:**
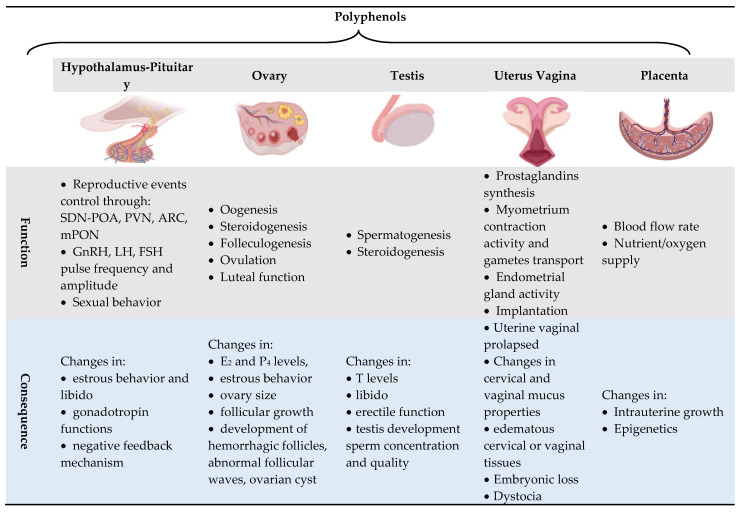
Effects of polyphenols on functions of reproductive organs and its consequences. Abbreviation: sexually dimorphic nucleus of the preoptic area (SDN-POA), paraventricular nucleus (PVN) arcuate nucleus (ARC), medial pre-optic area (mPOA), gonadotropin-releasing hormone (GnRH), luteinizing hormone (LH), follicle-stimulating hormone (FSH), estradiol (E_2_), progesterone (P_4_), testosterone (T). Changes refer to: positive or negative effects [11,20].

Therefore, polyphenolic compounds, due to their versatile biological functions, can affect reproductive functions at different levels and almost overlap with all in vivo reproductive events and intervene on the success of in vitro ARTs, as summarized in Figure 2 and discussed in the next sections. 

## 5. Polyphenols and In Vivo Reproductive Events

Summary of recent in vivo studies on the effects of polyphenols on the reproductive performance of males and females of farm animals is shown in Table 2 and Table 3. In brief, these studies address that farm animals consuming diets based on seeds and/or forage with polyphenolic compounds of isoflavones and lignans (phytoestrogens) evidence substantial reproductive disturbances in sexual activity, hormonal balance and gonads function (*T. pratense* L., red clover, silage: [11]; *T. alexandrinum*, berseem clover: [13,59]; *Glycine max,* soybean, or *Linum usitatissimum* linseed-based diets: [44,45]; linseed: [57]), which mask any positive effects on antioxidant activity and redox homeostasis [44,45]. These negative effects on reproductive performance are caused by the hormone-like effects, either acting as estrogen agonist and/or antagonist, of the polyphenolic compounds known as phytoestrogens (flavonoids, lignans, and stilbenes). 

On the other hand, feeding with tannins seems to be safer and even positive for reproductive events (*Quercus hartwissiana*: [70]; *Sesbania sesban*: [25]; *Acacia saligna*: [71]; *Punica granatum* seed: [66]). Conversely, the tannins group has further biological features than other polyphenolic compounds. Tannins, in excess, bind to dietary proteins and decrease protein adsorption through enterocytes, which can drive reproductive waste, such as decreasing ovulation rate and increasing embryonic loss [19]. However, moderate consumption can improve protein metabolism by increasing amino acids absorption and decreasing urea release [46]. Such improvement of the nutritional status of the animals exerts a positive effect on their reproductive performance, as evidenced in a trial [72] comparing ewes grazing *L. corniculatus* (tanniferous pasture) had higher ovulation and lambing rates than those grazing perennial ryegrass/white clover pasture (phytoestrogenic pasture). 

These data support the usefulness and relative safety of tanniferous plants (shrubs and trees), if properly consumed, for improving reproductive performance of animals (*Yucca schidigera*: [73]; Quebracho tannins: [12,74]). On the other hand, caution should be paid when breeding animals are fed phytoestrogenic polyphenols-rich (flavonoids, nonflavonoids, and phenolic acids) plants such as soybean, linseed, clover, milk thistle seeds, rosemary leaves [67], soybean isoflavones supplement [45], and green tea powder [5]. Indeed, bioavailability of tannins seems to be more restricted due to their high molecular weight and weaker absorption through the intestine, which may limit their effects [75]. 

Special remarks have to be made on the use of polyphenolic compounds for improving reproductive performance of farm animals challenged by stressful conditions. Supplementation with dietary propolis (a polyphenol-rich feed additive [76]) or *Moringa oleifera* (moringa) leaves ethanolic extract [68] improved redox status and semen quality traits of heat-stressed rabbit bucks while, in other study, propolis mitigated reproductive toxicity of cypermethrin in female rabbits [43]. Supplementation with wine *Punica granatum* (grape) pomace has also shown to improve redox status, testis weight, and semen quality traits of ram lambs kept under restrained conditions [69]. The enhancement of reproductive performance in these studies was related to the antioxidant activity of polyphenolic compounds and its ability to mitigate the harmful action of reactive oxygen species. These results support the protective role of polyphenolic compounds under stressful conditions, when harmful pathways such as oxidative and inflammatory stresses are activated. Thus, it could be concluded that polyphenolic compounds may lead to reproductive gains in stress-challenged animals. However, there is not enough information about the effects of the different polyphenols on reproduction and more research is needed to confirm or reject these assumptions, with emphasis on the polyphenol profile of each plant.

### 5.1. Sexual Behavior

Sexual behavior is a critical reproductive event, since it can be considered the initial step for the later cycle of the reproductive events and the lack or weakness of behavioral estrous signs in the females or libido and sexual behavior in the males lead to maintenance of animals without actual reproductive contribution to the herd, leading to reproductive and economic wastes. 

Several studies have confirmed the ability of polyphenols to affect sexual activity in different farm animals. Consumption of the shrub *Sesbania sesban* L. exerts a negative effect on the expression of behavioral estrus in sheep [77] and feeding *T. alexandrinum* L. isoflavones (212,076.2 μg/ewe/day rich in biochanin A) also prevents or shortens behavioral estrus in sheep [13,58]. Similarly, feeding a linseed-based diet (containing primarily secoisolariciresinol and daidzein), soybean-based diet (containing mainly genistein and daidzein) or soybean isoflavones (containing a ratio of 1 genistein: 5.7 daidzein) have been found to decrease libido of rabbit bucks [44,45]. Conversely, Mustonen et al. [11] reported that feeding sheep with *T. pratense* L. silage (containing formononetin, biochanin A, genistein, and daidzein) for a total of 5 months did not affect mean numbers of estrus per pregnancy (2.1 ± 0.7 for the *T. pratense* L. vs. 2.2 ± 0.8 grass silage in controls). 

These different results on the effects of polyphenols on sexual behavior may be explained by different mechanisms, but mainly by antiestrogenic effects that can interfere with endogenous estrogen action [13,77]. In any case, the effects of polyphenols depend on the concentration of endogenous E_2_ because polyphenols and E_2_ are competing for the binding sites on ERs and polyphenols may act as estrogenic antagonists, inhibiting the full estrogen activity by occupying a part of the ERs. Moreover, many polyphenols have a greater affinity for ERβ than for ERα, which is suspected to be involved in the antagonistic action of E_2_ [62]. There are also studies in males addressing that binding of polyphenols with ER in the brain causes changes in reproductive, stress-related, social behavior, and cognitive function. For example, the sexually dimorphic nucleus of the preoptic area (SDN-POA) is involved in the control of gonadotrophin release and the sexual behavior in males and the size of SDN-POA has been found to be associated with sexual partner preference [78]. In this regard, consumption of soybeans decreases the volume of the SDN-POA in males but not in females, compromising sexual and copulatory behavior. 

### 5.2. Hormone Secretion and Function 

Hormones are the critical signals for reproductive events and therefore adequate hormonal secretion and function play a crucial role in the success of such reproductive event. Several studies have revealed the ability of polyphenols to alter the biosynthesis and function of reproductive hormones along the hypothalamus-hypophysis-gonadal axis. 

At the brain level, polyphenolic compounds can affect sexually the dimorphic nucleus of the hypothalamus, controlling both sexual behavior and gonadotropin-releasing hormone (GnRH) (i.e.,: preoptic area, SDN-POA, paraventricular nucleus, PVN, arcuate nucleus, ARC, and medial pre-optic area, Mpoa [11,20]) and can distress the function of the pituitary gland and thus synthesis of both LH and FSH [20]. There are data addressing that infusion of genistein into the brain of ovariectomized ewes results in decreased LH-pulse frequency and plasma LH concentrations, while long term consumption of soy diets (containing genistein and daidzein) inhibits the LH-stimulated secretion of progesterone [79]. These disturbances in LH-pulse frequency and amplitude are associated with significant reductions in peripheral progesterone concentrations during the luteal phase and the early pregnancy of sheep [44] and heifers [59]. Conversely, other studies from Cools and co-workers [80] and Watzkova and co-workers [81] address that feeding soybean-isoflavones (genistein and daidzein) are not associated to decreases in peripheral P_4_ concentrations. A possible explanation for these inconsistent results may be found after in vitro studies of the effects of isoflavones (biochanin A and genistein) on progesterone synthesis by the granulosa cells of cattle; such effects are dose-dependent and biphasic; since P_4_ synthesis is stimulated by low doses and, conversely, doses above a threshold suppresses P_4_ synthesis [82]. 

At the gonads level, polyphenols can affect steroid synthesis by either ovarian granulose cells or testicular interstitial cells, due to their ability to alter the sensitivity of these cells to the action of gonadotrophins or the activity of enzymes involved in sex hormone biosynthesis. For example, the bark of condensed tannin-rich trees (e.g.,: *Acacia mangium*, *Sonneratia caseolaris*, *Acacia mearnsii*, *Salix rorida*, *Larix leptolepis*, *Cryptomeria japonica* and *Thujopsis dolabrata* var. *homadae*) can inhibit the activity of the steroid 5α-reductase by binding to the enzyme [83]. Isoflavones, stilbenes, and coumestans can inhibit 5α-reductase, aromatase and the 3β-hydroxysteroid dehydrogenase/isomerase complex by decreasing gene expression and/or inhibiting the enzyme itself [57,84]. In other studies, gossypol inhibits activity of 5α-reductase and 3α-hydroxysteroid dehydrogenase enzymes [85], as well as the antioxidant enzyme superoxide dismutase, via the second messenger cGMP pathway, leading to inhibition of steroid production by different molecular pathways [85]. Similarly, green tea extract can alter ovarian functions and blockade ovulation in rabbits. This is ascribed to its main constituent EGCG with proapoptotic and antisteroid hormone properties [5].

At the uterus level, isoflavones and their metabolites, the most abundant phytoestrogens derived from soybean, has been found to alter PGF_2α_ and PGE_2_ secretion in the bovine endometrial tissue, leading to increased PGF_2α_/PGE_2_ ratio which, in turn, may increase the possibility of luteolysis, implantation failure and, consequently, early embryo mortality or abortion [54]. However, an increased PGF_2α_/PGE_2_ ratio may be useful for luteolysis and ovulation of postpartum anestrous dairy cows, facilitating resumption of post-partum cyclicity. In this context, polypepigallocatechin-3 gallate has been also found to stimulate the activity of prostaglandin G/H synthase-2 (PGHS-2; also known as COX-2) and thus PGF_2α_ synthesis, which could be of interest for ovarian cyclicity when higher progesterone levels are maintained, hampering normal reproduction activity [86].

In some studies, these hormonal imbalances were associated with apparent reproductive disorders like, in females, increased risks for nymphomania, vaginal prolapse and labor difficulties [11], early embryonic loss [13], and reduction in fertility/fecundity [59] and, in males, low libido, erectile dysfunction, and oligospermia [20,44].

### 5.3. Gametogenesis

In both males and females, gametogenesis is a complex biological process that encompasses a serial of cellular divisions accompanied with structural (differentiation) and functional configurations. The progression of these processes is strictly controlled by hormones, mainly hormones of the *hypothalamic*–pituitary–*gonadal axis* and many cellular signaling factors [87]. Therefore, the implication of polyphenols in amending the efficiency of gonadogenesis is strongly expected. In adult animals, feeding either a soybean-based diet (containing genistein and daidzein isoflavones) or linseed-based diet (containing secoisolariciresinol lignan) to adult rabbits reduced significantly testosterone synthesis, the spermatogenic process, and libido. These effects seem to be stronger for linseed lignans than that of soybean isoflavones. Evans et al. found that enterolactone (a secoisolariciresinol metabolite) has the highest inhibition potency (98%) on 17 b-hydroxysteroid dehydrogenases activity compared with genistein (82%) and daidzein (34%). In addition, both isoflavones and lignans can activate ER pathways, depending on their relative binding affinity to ER and relative transcriptional potencies [88]. Given these findings, it could be suggested that lignans, mainly secoisolariciresinol, might have greater binding affinity or transcriptional potency than isoflavones, mainly genistein and daidzein, in adult male rabbits, leading to stronger biological actions on spermatogenesis, steroidogenesis, and libido. Additionally, Hadadi et al. [89] reported that the consumption of *Medicago sativa* (alfalfa) by adult rats for 30 days resulted in a positive transient effect on the number of seminiferous tubules, primary spermatocytes, sperm cells, and Leydig cells. However, when the experimental period was extended for 60 days, a reduction in the number of germ cells and Leydig cells was observed. It is known that polyphenols of alfalfa have a phytoestrogenic activity with estrogenic or antiestrogenic, antioxidant, and endocrine effects [90]. Phytoestrogen can reduce the proliferation of Leydig precursor cells presumably by activating macrophages phagocytosis [91].

Lifelong (from conception to adulthood) exposure to soybean-based diets containing 150 ppm daidzein and 190 ppm genistein reduced the number of haploid cells in the testis and epididymal sperm cells, without altering serum testosterone levels or Sertoli cells maturation and function of CD-1 mice. In this study, all markers that cover the different stages of early and midspermatogenesis remain unchanged [92]. However, the spermatid specific marker Gapd-s (glyceraldehyde 3-phosphate dehydrogenase-s), which encodes for a protein that regulates glycolysis, and thus sperm motility and fertility, was downregulated in the testis. Additionally, androgen receptor regulated genes were downregulated. These data suggest that the late stages of the spermatogenesis process, after round-spermatid stage, are the most sensitive stages to dietary polyphenols, which might be ascribed to the interference of soybean polyphones with the androgen receptor pathway. In context, male rats chronically exposed to genistein have abnormalities in spermatogenesis, resulting in alterations in sperm motility and a reduction in litter size accompanied by evidence of postimplantation embryo loss when the adult rats were subjected to fertility testing [93]. In females, there is growing evidence that polyphenols can affect oogenesis process at different levels. Polyphenols can affect the prenatal, postnatal and adulthood follicular development, controlling ovarian follicular/oocytes reserve, oocyte development, and quality [94,95].

On the other hand, positive effects of polyphenols such as resveratrol on gametogenesis process were observed under stressful conditions such as exposure to environmental toxins, high physical activity [96], and high oxidative stress [65] in both males and females.

### 5.4. Pregnancy and Fetal Programming

Inadequate developmental traits of the offspring are increasingly described in the last few years, due to the rising pressure for cost-efficient production (shortening nonproductive periods and increasing number of offspring per litter) or due to environmental challenges (nutrition, temperature, water availability, stress). The consequence of such perturbations is an increasing incidence of offspring affected by intrauterine growth restriction and, afterwards, low birth-weight. The occurrence of low birth-weight compromises viability and health of the neonate but, moreover, induces deleterious lifelong effects due to epigenetic changes.

Polyphenols are increasingly used in the maternal diets to counteract oxidative stress, low-grade inflammation and metabolic disturbances which are usually found in fetuses of compromised pregnancies. The use of polyphenols during gestation is based on its perception of “natural and nonharmful” products but there is very little knowledge on their effects when consumed by pregnant females and on the equilibrium between beneficial effects on development and metabolic traits of the fetus and deleterious effects due to their xenoestrogenic and epigenetic properties.

In this regard, flavonoids are possibly the most consumed polyphenols because they are found in most of herbs, fruits, and vegetables and, among them, quercetin is possibly the most consumed flavonoid. After quercetin, the intake of the isoflavone genistein is becoming high worldwide due to the increasing consumption of soy products as an alternative source of proteins. 

Hence, most of the research on the use of flavonoids during pregnancy has been performed using quercetin. Maternal supplementation with quercetin during pregnancy has been related to improvements of the maternal antioxidant/oxidative and metabolic status [97,98] and subsequent positive effects on fetal antioxidant capacity and developmental traits. These effects persist during postnatal stages, improving antioxidant defense systems and postnatal metabolic traits of the offspring, and decreasing oxidative stress-induced DNA damage [99,100,101,102,103]. Similar effects have been found with soy genistein-rich diets; specifically decreased oxidative stress and beneficial effects on the cardiovascular system, lowering incidence of hypertension [104]. The stilbene resveratrol has comparable positive effects on oxidative stress and metabolism in adults [105,106] and there is experimental evidence that ameliorates embryonic oxidative stress and apoptosis associated with diabetic embryopathy, preventing developmental delays in embryos of diabetic dams and, afterwards, glomerular loss and renal immaturity in fetuses [107,108,109,110]. 

However, exposure to high doses of flavonoids has been linked to pregnancy failures and epigenetic effects on offspring due to their role as xenoestrogens, as phytoestrogens. In fact, the most potent phytoestrogens are polyphenols (genistein, daidzein, and coumestrol [20]) and this aspect should be take into account when promoting intake of soy-derived products [111]. Exposure to phytoestrogens during prenatal development and early postnatal life can have marked effects on the reproductive system of the offspring [95], by disrupting sexual differentiation and reproductive function in adulthood. These effects are most abundantly described for genistein, although there is also evidence that xenoestrogenic effects of high doses of quercetin may affect embryo implantation and therefore pregnancy success [112] and, afterwards, may accelerate postnatal reproductive development in female offspring [113]. The exposure to high doses of genistein has been related to epigenetic changes [114,115,116] in genes modulating metabolism and adiposity, reproductive function [117], and appearance of different tumors [111,118,119,120]. 

Polyphenols, and specifically quercetin, are also potent modulators of low-grade inflammation by inhibiting cyclooxygenase expression [121] and acting on the conversion of fatty acids to prostanoids (prostaglandins, prostacyclins, and thromboxanes, with prostacyclin I2, PGI2, and thromboxane A2, TXA2, being the main products). The PGI2/TXA2 balance is critical for an adequate regulation of maternal and fetal vascular function during pregnancy [122]. Quercetin increases the ratio and may improve feto-placental circulation and some adverse outcomes of pre-eclampsia, such as neonatal death rate, although without significant improvements in the birth-weight of the neonate [123]. However, a highly increased ratio during the late pregnancy may trigger changes in the fetal vascular system; specifically, constriction of the *ductus arteriosus* which drives the fetal blood flow from the right ventricle to the descending aorta [124,125,126]. The constriction of the *ductus arteriosus* may cause fetal and neonatal heart failure, hydrops, and neonatal pulmonary hypertension, leading to death. Concomitantly, prenatal exposure to high doses of quercetin has been related to increased expression of inflammation associated cytokines at adult age [127]. Finally, quercetin, but also genistein and other polyphenols, inhibits the transport across the placenta of several bioactive compounds (organic cations, glucose, and vitamins B and C [128,129]. 

## 6. Polyphenols and In Vitro Assisted Reproduction Techniques

Summary of recent in vitro studies on the effects of polyphenols on the outputs of some ARTs in males and females of farm animals are shown in Table 4 and Table 5. 

It is clear that the reputation of polyphenols as strong antioxidant candidates encourages its inclusion in the media fabricated for in vitro manipulation of gametes/embryo culture, maturation, and preservation. The results of the studies shown in Table 4 and Table 5 support that polyphenols act as antioxidant agents, and thus most studied variables and result interpretations were based on this assumption. The antioxidant capacity of many polyphenols has been confirmed, including grape seed procyanidin extract [133], milk thistle silibinin [67], hydroxytyrosol and 3,4 dihydroxyphenylglycol [132], epigallocatechin-3-gallate and genistein [14], and resveratrol. A large body of evidence supported the potential of polyphenols to improve gains/outcomes of some ARTs.

Substantial improvements in preserved (liquid or freezing storage) semen quality traits and sperm fertilization [130,131,133,134], oocyte development and competence, and embryo development [15,16,139,140] have been reported. The other evidence that could be observed from in vitro studies is that supplementation of polyphenolic compounds during gametes or embryo in vitro manipulation might continue to in vivo animals. This adds an additional value for the inclusion of these compounds to the media of gametes/sperm manipulation. Ahmed et al. [130] found that addition of resveratrol (100 μg/mL) to frozen-thawed extender of buffalo semen significantly increased postartificial insemination pregnancy rate. Similarly, Wen et al. [133] found that addition of grape seed procyanidin extract (30 μg/mL) to extender of liquid-stored goat semen significantly increased litter size postartificial insemination. 

As discussed in different studies, these enhancements were mainly due to the protective role of polyphenols on cell membrane integrity, mitochondrial membrane potential, and DNA fragmentation against raising reactive oxygen species. It is widely believed that polyphenols can effectively modulate redox status of biological systems via different mechanisms. Polyphenolic compounds have the ability to directly scavenge reactive oxygen species such as superoxide anion radicals and hydroxyl radicals, thus interrupting free radical chain reaction. They also have the ability to enhance the expression and the activity of antioxidant enzymes throughout different pathways such as the nuclear factor erythroid 2–related factor 2 (Nrf2) signaling pathway [132,133]. However, it is worthy to note that polyphenols may play a reverse role and shift the redox reactions toward production of reactive oxygen species. This effect mainly depends on the level of polyphenol; high levels of polyphenol may massively scavenge reactive oxygen species or act as prooxidants by reducing metal ions, leading to the generation of free radicals [53,68]. Actually, however, reactive oxygen species are known as harmful molecules, their moderate levels are essential for completion of many biological functions in cells including gametes and embryos [53,132,139]. These facts explain the negative effects observed in some studies due to addition of high levels of polyphenols. For instance, bovine oocytes cultured in maturation medium supplemented with 15 μM/mL green tea polyphenols (99% catechin) derivatives, mainly EGCG, have higher intracellular glutathione (GSH) concentrations, maturation and cleavage rates, and blastocyst rates than those supplemented with 20 μM/mL [15]. In agreement with these observations, it has been stated that supplementation of a lower EGCG concentration (10 μg/mL) during in vitro fertilization significantly increased fertilization rate, while higher EGCG concentration (25 μg/mL) decreased fertilization rate [64]. The same observations were reported for ARTs applied for semen/sperm, as high polyphenol levels had detrimental effects on sperm membrane integrity and mitochondrial membrane potential, sperm capacitation, sperm motility, and sperm-zona pellucid binding and thus in vitro fertilization outcomes [132,135,138,139]. Overall, the action of polyphenols are associated with their chemical structure, level, cell sensitivity, and half-time of reactive oxygen species, which might explain different results obtained following using polyphenolic compounds as antioxidant agents [132].

Despite the enhancements in the outputs of applied ARTs due to antioxidant effects of polyphenolic compounds, other studies reported the ability of some polyphenolic compounds (epigallocatechin-3-gallate, genistein, biochanin A, formononetin) to inhibit proliferation of granulose cells [3], embryo cleavage rate [3,141], steroidogenesis [5,64], vascular endothelial growth factor (VEGF) synthesis [3], and accumulation of apoptosis-related factors such as caspase 3 [5]. These hazards refer to the potential of polyphenolic compounds to affect gene expression at very early stages of gametes/embryos development which might affect the completion of reproductive events such as pregnancy and the reproductive performance of offspring in the future. Thus, in vitro studies directed to test effects of polyphenols on ARTs outcomes should substantially consider the wide range of polyphenolic compounds biological activities rather antioxidant activity. This is important to draw an integrated overview for the effects of these compounds on the reproduction of farm animals and to ensure their safety to all reproductive events and animals’ performance, before being recommended as natural and safe chemical compounds.

It is worthy to note that in vitro studies testing polyphenolic compound effects on reproduction were not only carried out as an attempt to improve assisted reproductive technique outputs, but also as a tool to evaluate the relevance of new plant species on reproductive events [14,141]. Regardless of the positive or negative results of such studies, it should be noted that the nature of these studies does not consider the complexity of metabolic processes and bioavailability of polyphenols and their metabolites on the physiological and hormonal status of animals, and variations in plant composition. These factors can often cause changes in the effects of dietary polyphenols in vivo, leading to improper evaluation to the safety of the tested plant species. 

Overall, before applying the results of in vitro experiments on field scale, more details should be obtained regarding the reproductive performance of animals that have received gametes/embryos treated previously with polyphenolic compounds to ensure a lack of negative effects on reproductive performance of adult animals. Additionally, the vitality and future reproductive performance of resultant offspring should be monitored to ensure that there are no long-term negative effects that may affect the reproductive performance of the herd.

## 7. Polyphenols of Animal Origin and Human Health

In human diets, the main source of polyphenols are plants (fruits, vegetables, seeds, and grains), but animal products (milk and meat) may be also a potential source of polyphenols [142]. Thus, it is important to know the possible effects of polyphenols of animal origin on human health and reproduction. Polyphenols in animal products are derived from polyphenol-rich feeds and pastures (white clover, red clover, lucerne, and chicory-rich pastures [143]). Additionally, mammalian polyphenol metabolites such as enterolignans and equol are identified in animal products due to the action of gut microflora [144]. Milk of dairy cows and goats has been found to contain different polyphenolic compounds, including lignans, coumestans, and isoflavones (genistein, daidzein, formononetin, and biochanin A), as well as the isoflavone equol (a metabolite of formononetin [144,145,146]).

In bovine milk, the concentration of polyphenols is described to be around 132.4 mg/L [147]. Considering the polyphenol class, the concentration of the isoflavones (genistein, daidzein, biochanin A, and formononetin,) have been found to range from 0.1 to 7.7 mg/L, the lignan enterolactone between 19 and 96 mg/L, and the concentration of equol between 45 and 364 mg/L [148,149,150]. These data show the prevalence of the polyphenol equol in milk. It is assumed that the content of polyphenols in the animal products is lower than that in the plants (e.g., soybean and their products [142]); however, polyphenols of animal origin are of particular importance due to the presence of considerable concentrations of mammalian polyphenols, which could not be directly obtained by consumption of plants as their synthesis depends on the action of human and/or animal gut microflora. In this term, only 20–35% of the adult human population is believed able to produce equol [151], whereas animal populations commonly produce equol after ingesting isoflavones. There have been different studies on the potential benefits of mammalian polyphenol on human health, specifically equol, suggesting a prominent role in decreasing the risk of cardiovascular diseases, diabetes, neurodegenerative diseases, tumor development, osteoporosis, and menopause-related symptoms [152,153,154]. On the other hand, these compounds also pose risks to human health because of their capacity to affect both the antioxidant status and the endocrine system. Particularly, mammalian polyphenols have a higher estrogenic potency than their original precursors [29,44].

To our knowledge, there are few studies on the effects of the consumption of polyphenols of animal origin on human reproductive health. Most of the studies in such field focused on the effects of plant polyphenols, mainly soybean isoflavones, on human reproduction during prenatal, childhood, and adulthood periods [51,90,92,94]. The main conclusion of these studies is that the consumption of isoflavones is related with many reproductive disorders, such as advancement of the menarche [155], sexual dimorphic behavior such as masculinization [156], and malformations of the reproductive tract [157,158]. Other groups of studies that indirectly investigated the effects of polyphenols from animal origin has been devoted to differentiate the effects of feeding soybean-based formula, cow milk, and maternal breastfeeding on reproduction functions pre- and postadulthood [103,150,159,160]. In this context, Harlid et al. [161] studied the effects of feeding soybean-based formula and cow milk on DNA methylation levels in vaginal cells of newborn infants. Results showed that methylation levels of the gene proline rich 5 like (PRR5) decreased with increasing age but soy formula-fed girls maintained higher mean methylation levels compared with cow formula-fed girls. The suppression of PRR5L expression has been suggested to promote tumor necrosis factor alpha (TNFα)-associated autoimmune diseases, such as asthma, rheumatoid arthritis, psoriasis, and inflammatory bowel disease at adulthood [162]. 

## 8. Conclusions

The present review presented the current information on the relationship between dietary polyphenols and reproductive events in farm animals, aiming to help discern whether polyphenols are a source of reproductive gain or waste. The assessment of the current information supported that biological activity and risk/benefit of polyphenolic compounds are dependent on their diversity, dual-effects, biological activity, and source. On the other hand, animal species, age, sex, physiological and health status, timing and length of exposure, polyphenols metabolism, and bioavailability are other physiological factors modulating their effects.

These factors have to be in mind when applying polyphenols to farm animals. Polyphenols, being “natural” products, are perceived as beneficial and nonharming products. However, the analysis of the currently available information evidences a great gap in our knowledge regarding the safety of these compounds to reproductive processes. Overall, the presence of polyphenols in the diets of farm animals during their reproductive cycle and/or in the ARTs media can improve their reproductive performance when used properly; however, there are still associated unknown reproductive hazards. Thus, more research, about the profile of active polyphenolic compounds in each plant and about the biological effects of each polyphenolic compound, is needed before using new plant species, agro-industrial byproducts, and/or polyphenols-rich diets in breeding animals.

## Figures and Tables

**Table 1:** Sources of polyphenolic compounds in feed ingredients of farm animals.

Dietary Source (Reference)	Total Phenols, mg/g DM	Majorclass	Identified Polyphenolic Compounds
**Feed concentrates**			
*Glycine max* [21]	2.23–6.18	Isoflavone	Genistein, daidzein, glycitein, formononetin, puerarin, coumestrol
*Linum usitatissimum* [22]	8.0–10.0	Lignans	Secoisolariciresinol, matairesinol, pinoresinol, pinoresinol, isolariciresinol, esterified phenolic acids, Kaempferol, herbacetin
**Forage and shrubs**			
*Trifolium subterraneum* [17]	24.2–114.2	Flavonoids	Flavonoids, such as flavones, flavonols and isoflavones
*Trifolium pratense* L. [11,18]	14.82	Isoflavones	Formononetin, biochanin A, genistein, daidzein, ononin, sissotrin, phenolic acids
*Trifolium alexandrinum* [18,23]	52.55	Isoflavone	Biochanin A, formononetin, genistein, daidzein, ononin, sissotrin, clovamides (caffeic acid derivatives), phenolic acids
*Medicago sativa* [18]	70.50	Coumestans	Coumestans, formononetin, biochanin A, genistein, daidzein, ononin, sissotrin
*Sesbania sesban* [24,25]	167.66	Tannins, phenolic acids	Gallic acid, catechin hydrate, vanillic acid, caffeic acid, epicatechin, rutin ellagic acid, myricetin, kaempferol, quercetin
*Moringa oleifera* [26,27]	96.30	Flavonols, phenolic acids	Caffeoylquinic acids, carotenoids, lutein, kaempferol, quercetin, ellagic acid, and apigenin glucoside, myricetin, rutin, isorhamnetin, ferulic acid, coumaric acids, caffeic acid, gallic acid, ellagic acid, chlorogenic acid, epicatechin
*Lotus corniculatus* [28]	-	Flavonoids	Kaempferol, quercetin, isorhamnetin and their derivatives
*Cichorium intybus* [29]	0.65–3.73	Flavonoids	Chlorogenic and caffeic acids
*Salix* spp. [30]	212.0	Tannins	Condensed tannins, salicylates, cinnamic acid derivatives, gallic acid, caffeic acid, vanillin, p-coumaric acid, myricetin, catechin, epigallocatechin gallate, rutin, quercetin, salicin
*Acacia etbaica* [31]	-	Tannins	Proanthocyanidin, galloyl derivatives kaempferol, quercetin, myricetin
*Quercus robur* [32]	25.30–50.95	Tannins	Ellagitannins (roburin A,B,C,D,E; grandinin, vescalagin; castalagin), protocatechuic acid/aldehyde, gallic acid, vanillic acid, caffeic acid, vanillin, syringaldehyde, coumaric acid, scopoletin, ferulic acid, sinapic acid, coniferyl aldehyde, sinapaldehyde, ellagic acid
**Agro-industrial byproducts and feed additives**			
*Vitis vinifera* pomace [33,34]	14.8–70.5	Tannins, anthocyanins	Anthocyanins, condensed tannins, catechin, epicatechin, gallic acids, cyanidin 3-glucoside, malividin 3-glucoside, cyaniding, and peonidin, resveratrol
*Olea europaea* L. cake [35]	4.1–19.4	Flavanone	Tyrosol, hydroxytyrosol, oleuropein, verbacoside, rutin, luteolin, apigenin, quercetin
*Citrus sinensis* peel [36]	104–223	Flavanone	Hesperidin, quercetin, eriocitrin, narirutin, isosakuranetin rutinoside, kaempferol, gallic acid, ferulic acid, p-coumaric, catechins, epicatechins
*Punica granatum* seed [37]	27.2	Tannins	Flavonoids, anthocyanins, hydrolysable tannins, gallic acid
*Punica granatum* peel [38]	48.3	Tannins	Gallic acid, punicalagin, punicalin, flavonoids, hydrolysable tannins, condensed tannins
*Solanum lycopersicum* [39]	6.1–6.4	Flavonols	Naringenin, rutin, quercetin, kaempferol
*Citrus aurantifolia* [36]	104–223	Flavanone	Hesperidin, quercetin, kaempferol, gallic acid, ferulic acid, p-coumaric, catechins, epicatechins,
*Camellia sinensis* [40]	148.16–252.65	Catechins	Catechin, epicatechin, gallocatechin, epigallocatechin, catechin gallate, epicatechin gallate, gallocatechin gallate, gallic acid, ellagic acid, quercitrin, astragalin, quercetin, kaempferol, chlorogenic acid, myricetin
*Phoenix dactylifera* pits [41]	12.7–47.7	Phenolic acids	Hydroxytyrosol, tyrosol oleuropein, gallic acid, ferulic acid, coumaric acids, p-hydroxybenzoic acid, flavonoids
Propolis [42,43]	65.49–228.4	Phenolic acids, flavonoids	Gallic acid, caffeic acid, catechin, chlorogenic acid, p-coumaric acid, ferulic acid, naringenin, quercetin, apigenin, baicalin, luteolin, pinocembrin, galangin

**Table 2:** Summary of some recent in vivo studies on the effects of polyphenols on reproductive performance of male farm animals. IVF = in vitro fertilization.

Animal (Reference)	Treatment	Main Results
Barki rams [26]	0, 40 mg/kg diet *Moringa oleifera* leaves extract	Fresh semen: increased semen volume, sperm concentration, activities of seminal plasma catalase, glutathione peroxidase, glutathione reductase, superoxide dismutase, alkaline phosphatase, acid phosphatase levels of ascorbic acid, and total antioxidant capacity Frozen-thawed semen: increased post-thawing sperms motility, viability index, membrane integrity, and semen antioxidant enzyme activities. Decreased seminal plasma concentration of malondialdehyde, acrosomal defects, and DNA fragmentation.
Boar [47]	0, 2, 4% of diet *Vitis vinifera* marc	Both levels: Improved kinetic variables, membrane integrity of fresh and stored semen (refrigeration at 17 °C) Decreased sperm abnormalities and lipid peroxidation of fresh and stored semen
Lambs [57]	0,4, 8% of diet *Linum usitatissimum*	At 8%: Decrease testosterone and blood genomic DNA content Increased growth hormone and thyroid stimulating hormone
Rabbit bucks [45]	0,5, 20 mg/kg body weight *Glycine max* isoflavones (5 daidzein: 1 genistein)	At both levels: Decreased libido, sperm concentration, and testosterone Increased triiodothyronine At 20 mg/kg BW: Increased total antioxidant capacity and reduced malondialdehyde
Rabbit bucks [44]	*Glycine max*-based and *Linum usitatissimum*-based diets	Both diets: Improved sperm motility and viability Increased triiodothyronine Decreased libido, sperm concentration, and testosterone No effect on bucks’ fertility Improved total antioxidant capacity and reduced malondialdehyde
Cloned goat bucks [66]	0, 8.83, 17.66% of diet *Punica granatum* seed for 9 weeks	Increased sperm motility and vitality, cell membrane integrity of frozen-thawed semen No effects on cleavage rate and blastocyst development following IVF
Rabbit bucks [67]	0, 5, 10 g/kg of diet milk thistle seeds and rosemary leaves	At milk thistle seeds 10 and rosemary leaves at 5 g/kg diet: Increased sperm concentration, sperm vitality and motility Improved testosterone and fertility
Heat stressed rabbit bucks [68]	0, 50, 100, 150 mg/kg body weight (BW) *Moringa oleifera* leaves extract	All levels: Improved sperm quality traits High level (150 mg/kg BW) tended to decrease testosterone and total antioxidant capacity
Ram lambs kept under restrained conditions (pen conditions) [69]	0, 5, 10% of DM diet wine *Punica granatum* pomace for 74 days	Increased testes weight, sperm concentration, motility, and acrosomal integrity and testicular antioxidant status in pen-raised animals

**Table 3:** Summary of some recent in vivo studies on the effects of polyphenols on reproductive performance of female farm animals.

Animal	Treatment	Main Results
Rabbit does [5]	0, 5, 20 g/100 kg diet *Camellia sinensis* powder from weaning (45 days old) and throughout two consequent reproductive cycles	Increased ovarian length and diameter of ovarian nonovulated peri-ovulatory hemorrhagic but not of primary and secondary growing follicles. Reduced conception and kindling rate, the number of live-born and weaned pups Increased female mortality but not their weight gain
Buffalo cows [70]	0, 100, 200 g/head/day Quebracho tannins	No effects on progesterone levels, number of service per conception and conception rate
Seasonal anestrous Rahmani ewes [13]	*T. alexandrinum* vs. corn silage 2 weeks prepartum to 8 weeks post induced estrus	Progesterone reduction Shortened duration of estrus Suppressed the developmental capacity of small and medium follicles No effects on corpora lutea numbers and diameters Reduced fecundity and litter size
Prepubertal hair breed ewe lambs during the natural anestrous season [65]	0, 300 mg of ferulic acid/day/head for 34 days	Increased reproductive tract weight, ovarian mass Increased number of larger follicles and corpus luteum and percentage of ewe lambs with large follicles and corpora lutea No effect on number of small follicles and percentage of ewe lambs with small follicles
Barki ewes	Quebracho condensed tannins (20 g/head/day) for four weeks pre-mating to lambing	No effects on ovarian follicles number and size or corpora lutea numbers and diameter and progesterone and estradiol levels.
Barki, fat-tailed, Ewes [71]	50, 100% substitution of clover hay by tannins-rich plant (*Acacia saligna*)	No change in conception rates Improved fertility and lambing rates No differences in progesterone concentration
Rabbit does [73]	*Yucca schidigera* extract (0, 5, 20 g of Y powder extract per 100 kg diet for 350 days.	Enhanced plasma oxytocin and prostaglandin F_2α_ levels; Increased plasma progesterone concentration by low-dose *Yucca schidigera*, but decreased by high-dose *Yucca schidigera* Increased conception rate
Holstein heifers [59]	*Trifolium alexandrinum* vs. corn silage for five month, 3 premating plus two post-mating	Progesterone reduction, estradiol increase, high estradiol to progesterone ratio High return to estrus Increased no. of services/conception
Cypermethrin-challenged rabbit does [43]	50 mg/kg bodyweight propolis	Improved redox status Mitigated negative effects of cypermethrin on ovarian histology, steroid synthesis (progesterone and estradiol) and reproductive performance
Finnish Landrace ewe lamb [11]	*T. pratense* L. silage vs. grass silage for five month, 3 premating plus two post-mating	Increased total uterus mass Progesterone reduction No altered fecundity Increased urea level
Prepubertal female Tuj lambs [70]	*Quercus hartwissiana*	Not effect on gonadotropin-releasing hormone induced luteinizing hormone secretion in prepubertal female
Menz ewes [25]	0.28% of diet *Sesbania sesban* during pre-mating, mating, pregnancy and lambing	No effect on progesterone levels Improved conception rate by 17% Improved litter size

**Table 4:** Summary of recent in vitro studies on the effects of polyphenols on outputs of some assisted reproductive techniques (ARTs) in male farm animals.

ARTs	Source/Dose	Main Results
Semen frozen-thawed extender of buffalo [130]	0, 10, 20, 50, 100 µM/mL RES	At 50, 100 µM/mL: Increased sperm motility, antioxidant status (higher SOD, GPx, CAT) At 100 µM/mL: Decreased DNA fragmentation Increased sperm plasma membrane integrity and mitochondrial membrane potential and post-AI pregnancy rate
Liquid store (cooling at 5 ◦C) extender for 168 h of ram semen [131]	0, 200, 400 µM/mL RES	At 400 µM/mL: Increased progressive motility, antioxidant status (higher SOD and GSH, lower MDA) Protected sperm head morphology, improved kinematic parameters and in vitro fertility (cleavage and blastocyst rates)
Semen frozen-thawed extender of rams [132]	0, 10, 30, 50, 70 μg/mL of HT, DHPG and a mixture (MIX)	At all levels of HT and DHPG: Reduced LPO No change in sperm plasma membrane integrity, acrosome status, mitochondrial membrane potential MIX reduced sperm membrane, and acrosome integrity
Liquid storage (4 °C, 120 h) of goat semen [133]	0, 10, 30, 50, and 70 mg/L GSPE	At 30 mg/L: Increased sperm motility, acrosome and plasma Membrane integrity, mitochondrial activity, improved antioxidant status of semen (higher TAC, CAT, SOD, and lower MDA) Improved AI outcomes (litter size)
Frozen-thawed extender of goat semen [134]	0, 10, 50, 100, 250 μM/mL RES	At 10 or 50 μM/mL: Increased progressive motility, membrane and acrosome integrity, mitochondrial activity, and sperm viability Reduction in ROS
IVF medium of boar semen [135]	0, 1, 10, 100 µg/mL tannin (*Quercus robur*) and its four fractions (FA, FB, FC, FD),	At 10 µg/mL: Increased sperm capacitation and fertilization rate At 100 µg/mL: Suppressed capacitation and fertilization rate The highest potency was for FB fraction
Frozen-thawed extender of buffalo semen [136]	0, 0.5, 1, 10 and 50 µM/mL RES	At 50 µM/mL: Decreased capacitation-like changes, oxidative stress (higher TAC, lower ROS and MDA) Improving membrane stability and IVF ability No effects on 60 days pregnancy rate in vivo
Post-thawing semen extender of boar for 1 h [137]	0, 0.5, 1, 2 mM/mL RES or 0, 25, 50, 100 µM/mL EGCG	RES or EGCG did not affect sperm viability and acrosome integrity EGCG 25 and 50 µM and RES 2 mM increased fertilization rate
IVF medium using cryopreserved bovine spermatozoa [138]	0, 0.074, 0.74, 7.4 µmol/L GEN	No effect on sperm motility and capacitation At 7.4 µmol/L: inhibition of sperm-zona pellucida binding and reduced acrosome reaction

In vitro fertilization (IVF), resveratrol (RES), hydroxytyrosol (3,4-dihydroxyphenylethanol, HT), 3,4 dihydroxyphenylglycol (DHPG), grape seed procyanidin extract (GSPE), epigallocatechin-3-gallate (EGCG), genistein (GEN), superoxide dismutase (SOD), glutathione peroxidase (GPx), glutathione reductase (GSH), malondialdehyde (MDA) total antioxidant capacity (TAC), Catalase (CAT), reactive oxygen species (ROS), artificial insemination (AI).

**Table 5:** Summary of recent in vitro studies on effects of polyphenols on outputs of some assisted reproductive techniques (ARTs) in female farm animals.

ARTs	Source/Dose	Main Results
IVM of ewe oocytes [5,14]	Methanolic plant extracts: 0, 50, 100 mg/mL *Bituminaria bituminosa, Medicago sativa*, *Cichorium intybus, Trifolium (T.) subterraneum, T. pratense* L, *Biserrula pelecinus* and *Eremophila glabra*	At 100 mg/L: *B. pelecinus* improved fertilization and embryo development. *C. intybus* (50 and100 mg/mL) increased TCN but *M. sativa* (50 and 100 mg/mL) decreased TCN. Other plant extracts did not affect embryo cleavage or development rate
IVC of ovarian fragments from of rabbit does [5]	0, 1, 10 or 100 mg/mL EGCG, GTPP and RSV	EGCG increased accumulation of caspase 3, whilst both GTTP and RSV decreased EGCG inhibited both P_4_ and T output (10 or 100 µg/mL) GTPP stimulated P_4_ (10 or 100 µg/mL) and inhibited T (all doses) RSV promoted release of both P_4_ (1 µg/mL) and T (1 or 10 µg/mL).
IVM of bovine oocytes either supplemented (IVM A) or not supplemented (IVM B) with cysteine and b-mercaptoethanol [139]	0, 1, 5, 10, 20 µg/mL oenological *Quercus robur*-derived tannin	At all tested levels: No effect on oocyte nuclear maturation. Either in IVM A or IVM B No effect on E_2_ and P_4_ secretion by cumulus cells Improved antioxidant status in IVM B At 20 µg/mL: Reduced oocyte cytoplasmic maturation
IVM of prepubertal goat oocytes with high [16] (+) or low (−) quality	0, 1 µM/mL RSV	Oocytes (+,−) cultured in REV-containing IVM media had increased blastocyst production and GSH levels after IVF No effects on mitochondrial activity, ROS and ATP in resultant blastocysts
Exp 1: IVC, alone, of sheep embryos Exp 2: IVM of oocyte and IVC of sheep embryos [140]	0, 0.1, 0.25, 0.5, 2.0, 5.0 µM/mL RSV	Exp 1: At 0.25 and 0.5 µM/mL: Enhanced morula and blastocyst rates At 0.5µM: Increased numbers of trophectoderm and inner cell mass, and total cell number of blastocysts Exp 2: At 0.5 µM/mL Increased morula and blastocyst rates At 5.0 µM/mL:Decreased morula and blastocyst rates
IVM of oocytes and IVF in sheep [141]	0, 2.5, 5, 10, 25 µg/mL GEN, BIO A, FOR	At 25 µg/mL: GEN decreased cleavage rate, blastocyst rate and blastocyst efficiency BIO A decreased cleavage rate, blastocyst efficiency and total cell count hatched blastocyst stage FOR reduced blastomeres number of hatched blastocyst stage
IVM of bovine oocytes [15]	0, 10, 15, 20 μM/mL green tea catechin	At 15 μM/mL:increased cleavage and blastocyst rates and intracellular GSH concentration of oocytes
IVM and IVF of swine oocyte [64]	0, 2.5, 5, 10,25 µg/mL EGCG	At ≤10 mg/mL: No effect on oocytes maturation, oocytes degradation, fertilization and monospermy rates At ≥10 mg/mL: Inhibited spontaneous acrosome reaction At 25 mg/mL: Decreased parthenotes developed to blastocyst Decreased P_4_ synthesis but no effect on E_2_ Decreased percentage of fertilized oocytes
IVC of swine granulosa cells (From follicles >5 mm) [3]	0, 5, 50 µg/mL EGCG	Both levels: inhibited proliferation, steroidogenesis, VEGF production

In vitro maturation (IVM), in vitro culture (IVC), green tea polyphenols (GTPP) in vitro fertilization (IVF), epigallocatechin-3-gallate (EGCG), green tea polyphenols (GTPP), genistein (GEN), resveratrol (RSV), biochanin A (BIO A), formononetin (FOR), progesterone (P_4_), testosterone (T), glutathione reductase (GSH).
